# Non-diabetic hypoglycemia: bridging gaps in diagnosis and treatment: a position statement report by the Arabic Association for the Study of Diabetes and Metabolism (AASD)

**DOI:** 10.1186/s40842-025-00234-8

**Published:** 2025-10-06

**Authors:** Amin Roshdy Soliman, Rabab Mahmoud Ahmed, Amr Abdel Hady El Meligi, Ahmed Haroun Abdelmoaty, Mona A. Hegazy, Ahmed Saad, Sahar Alwakil, Nadine Alaa Sherif, Shereen Abdelghaffar, Ebtesam Fahmy, Saeed Soliman, Dina Farouk, Inass Shaltout

**Affiliations:** 1 Internal Medicine and Nephrology Department, Faculty of Medicine, Cairo University, Cairo, Egypt; 2 Internal Medicine and Nephrology Department, Kasr Alainy Faculty of Medicine, Cairo University, Cairo, Egypt; 3 Internal Medicine and Endocrinology Department, Faculty of Medicine, Cairo University, Cairo, Egypt; 4 Internal Medicine Department, Faculty of Medicine, Cairo University, Cairo, Egypt; 5 Internal Medicine Department, Hepatology and Gastroenterology Division, Faculty of Medicine, Cairo University, Cairo, Egypt; 6 Internal Medicine and Infectious Diseases Department, Faculty of Medicine, Cairo University, Cairo, Egypt; 7 Internal Medicine, Diabetes, Endocrinology, and Clinical Nutrition Department, Faculty of Medicine, Cairo University, Cairo, Egypt; 8 Obstetrics and Gynecology Department, Faculty of Medicine, Cairo University, Cairo, Egypt; 9 Pediatrics, Pediatric Diabetes and Endocrinology Department, Cairo University, Cairo, Egypt; 10 Neurology Department, Faculty of Medicine, Cairo University, Cairo, Egypt; 11 Family Medicine Department, Kasr Alainy School of Medicine, Cairo University, Cairo, Egypt; 12 Internal Medicine and Diabetes Department, Cairo University, Cairo, Egypt

**Keywords:** Nondiabetic, Hypoglycemia, Challenges

## Abstract

**Background:**

Non-diabetic hypoglycemia presents a substantial diagnostic challenge due to its diverse and often nonspecific symptomatology. The resultant delay in diagnosis can lead to a myriad of misdiagnoses. This misidentification not only prolongs patient suffering but also elevates the risk of severe complications, such as permanent neurologic damage and mortality. Recognizing the clinical nuances of non-diabetic hypoglycemia is paramount for timely and accurate diagnosis, which ultimately enhances patient outcomes and safety.

**Main Body:**

A position statement has been formulated by the Arabic Association for the Study of Diabetes and Metabolism (AASD) to address the complexities of diagnosing non-diabetic hypoglycemia. This statement highlights the challenges and pitfalls, covering key areas such as the vast spectrum of non-diabetic hypoglycemia within underreported subspecialties. It aims to elucidate the critical need for heightened clinical awareness and multidisciplinary management approaches to mitigate the profound impacts on patient health. Additionally, the statement provides guidance for overcoming barriers to optimal care. The team has distilled their experience into clinical algorithms to simplify information for healthcare professionals.

**Conclusion:**

Non-diabetic hypoglycemia is a rare condition that requires a comprehensive approach, including detailed history, medication review, and physical examination. Common pitfalls in diagnosis and management should be understood to avoid unnecessary evaluation and costs while avoiding misdiagnosis of patients with treatable or serious disorders.

## What is new in this manuscript?

1. This manuscript delves into the extensive realm of non-diabetic hypoglycemia within scarcely published subspecialties, including critical care medicine, pediatrics, obstetrics, neuropsychiatry, nephrology, hepatology, oncology, post-surgical care, and family medicine.
2. It synthesizes the collective expertise of a substantial interdisciplinary team to delineate diverse phenotypes of non-diabetic hypoglycemia. The manuscript critically analyses the multifaceted challenges in the evaluation and management of this condition, highlighting common pitfalls that may result in misdiagnosis or underdiagnosis, with potentially severe consequences.
3. The authors have integrated their extensive clinical experience into the development of clinical algorithms designed to simplify and enhance the utility of data for healthcare professionals.

## Introduction

Hypoglycemia is a serious medical condition, and although it is uncommon in people without diabetes, it is clinically relevant and often represents a challenge in diagnosis and management. It is usually underdiagnosed, resulting in delayed management affecting patient safety, quality of life, and potentially fatal consequences including permanent neurologic damage and death [1]. Hypoglycemia diagnosis is based on the classic Whipple’s triad. Typically, symptoms of hypoglycemia begin with palpitations, tremors, anxiety, and sweating due to sympathetic over activity, followed by neuroglycopenic symptoms due to brain glucose deprivation such as weakness, fatigue, confusion, unusual behavior, seizures, focal neurologic deficit, and coma [2]. However, in some individuals, neuroglycopenia occurs without preceding autonomic symptoms which adds to the challenges and difficulties in diagnosis [3]. Hypoglycemia in people without diabetes usually results from underlying disorders causing an imbalance between glucose utilization and glucose production or a combination of both [4]. Causes of hypoglycemia not related to diabetes include endocrine disorders, post-bariatric surgery complications, drugs, alcohol, end-organ failure such as renal and hepatic failure, sepsis, autoimmune hypoglycemia, and certain neoplastic conditions. Documentation of low plasma glucose levels during spontaneous hypoglycemic episodes or during supervised fasting tests, in addition to careful history taking, are crucial to confirm a clinical suspicion of hypoglycemia and to guide further investigations and decisions regarding the underlying cause [4]. The AASD has released this position statement addressing the complexities of diagnosing non-diabetic hypoglycemia, emphasizing the need for heightened clinical awareness and multidisciplinary management approaches to mitigate health impacts.

## Methods

The primary objective of this statement is to present a comprehensive review of the most relevant challenges in the evaluation and management of non-diabetic hypoglycemia. The scope includes many causes of hypoglycemia not related to diabetes. The main neuropsychiatric manifestations of hypoglycemia that sometimes mislead the diagnosis will be reviewed. To achieve this goal, the most reliable scientific evidence was utilized, while considering diagnostic challenges and treatment strategies.

The AASD offers practical recommendations for diagnosing and managing non-diabetic hypoglycemia, based on its experience, to avoid misinterpretation or underdiagnosis, ensuring a tailored approach for patients in various clinical settings.

A panel of thirteen experts from various medical fields, including diabetology, endocrinology, hepatology, nephrology, infectious diseases, clinical nutrition, obstetrics, pediatric endocrinology, neurology, and family medicine, was selected based on clinical experience, knowledge, and contributions to scientific research.

The position statement was based on a comprehensive evidence review, utilizing databases, keywords, and recent full-text publications, with expert panel review and full-text screening. Each expert provided input on draft recommendations based on search and evidence review. The consensus process was essential to ensure that the recommendations precisely represented the collective judgment of the expert panel.

### Non-diabetic Hypoglycemia in Endocrine Disorders

**Fig. 1:**
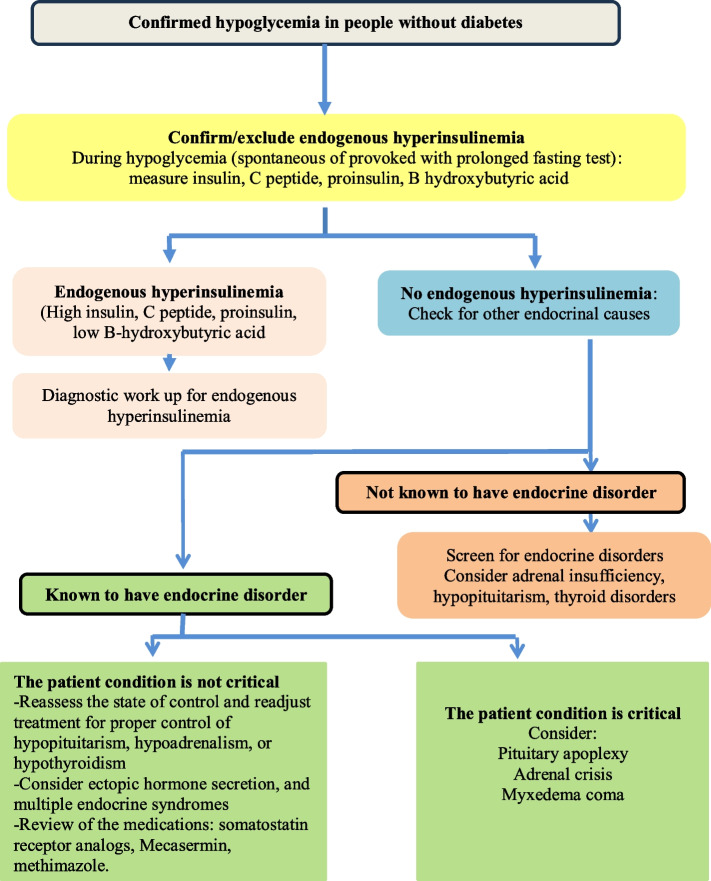
Algorithm demonstrating the approach to endocrine causes of non-diabetic hypoglycemia

It is classified as fasting or reactive, with or without hyperinsulinism (Fig. 1). *Fasting hypoglycemia (FH)* occurs during fasting (often nocturnal) or after skipped meals. FH with hyperinsulinism can result from insulinoma, exogenous insulin, insulin secretagogues such as sulfonylureas (SU), or drugs like pentamidine [5]. It is often linked to hyperinsulinism and requires careful diagnosis and management. Insulinoma is a rare β-cell tumor, with an incidence of 1–4 cases per million annually. Most tumors are pancreatic, with 3% being ectopic (often duodenal), 40% smaller than 1 cm, and 4% associated with multiple endocrine neoplasia type 1 (MEN-1) syndrome. It is slightly more common in women, typically presenting in the fifth decade. Symptoms include recurrent episodes of hypoglycemia, typically fasting or post-exercise, but in some patients, the symptoms may occur during both fasting and postprandial periods, or rarely, only postprandial [6]. Insulinoma patients may exhibit neuroglycopenic symptoms without autonomic signs, often misdiagnosed as neuropsychiatric disorders [7]. Patients frequently eat multiple meals and gain weight. Laboratory findings in insulinoma include high plasma insulin, C-peptide, and proinsulin, low β-hydroxybutyric acid (due to the antiketogenic effect of insulin), and negative urine/blood SU tests during confirmed hypoglycemia, either spontaneous or during a 72-h supervised fast test [6].

Factitious hypoglycemia due to surreptitious intake of SUs has a similar profile with positive urine/blood tests for SUs, while in exogenous insulin administration, there is high insulin, low C-peptide, and negative urine/blood SU tests. Factitious hypoglycemia is suspected in healthcare workers or those with psychiatric or social issues who have access to insulin or drugs, and the diagnosis is usually aided by identifying injection sites [8]. Given the small size of insulinoma, non-visualization with standard imaging tools does not rule out the diagnosis. If imaging fails to localize the tumor, selective arterial calcium stimulation with hepatic venous sampling is employed [9]. Medications that suppress insulin secretion, such as diazoxide, and somatostatin analogs (SSAs) are reserved for patients who refuse surgery, non-surgical candidates, or have non-localized tumors. Patients should be educated to consume frequent, slow-absorbing carbohydrate meals to prevent hypoglycemia and to recognize and manage early symptoms. Driving and heavy exercise are discouraged in patients with glycemic fluctuations, and continuous glucose monitoring (CGM) is recommended for improved management [6].

*Autoimmune hypoglycemia (AIH)* is a rare cause of postprandial hypoglycemia (PPH) with hyperinsulinism, involving insulin antibodies (insulin autoimmune syndrome, IAS) or insulin receptor antibodies (Type B insulin resistance, Hirata). IAS was first identified in Japanese patients with Graves'disease taking methimazole. Other causes include carbimazole, propylthiouracil, captopril and some autoimmune diseases. In IAS, early PPH occurs due to binding between anti-insulin antibodies and the insulin released after meals, forming immune complexes followed by hypoglycemia due to insulin dissociation from the immune complexes [10]. In Type B insulin resistance, insulin receptor-agonizing antibodies bind to insulin receptors, leading to PPH. Patients with this type have other features of insulin resistance, such as acanthosis nigricans [10]. Diagnosis of AIH involves detecting anti-insulin or anti-insulin receptor antibodies, with an insulin/C-peptide ratio greater than 1 distinguishing it from insulinoma, in which the insulin/C-peptide ratio is less than 1. Treatment includes dietary management, medications (e.g., SSAs, diazoxide, immunosuppressants), and management of underlying conditions. Mortality is linked to hypoglycemia and associated diseases like systemic lupus erythromatosus or cancer [10]

*Fasting hypoglycemia unrelated to hyperinsulinism* can occur due to endocrine gland dysfunction or surgical removal, often linked to deficiencies in anti-insulin hormones like growth hormone (GH), cortisol, thyroid hormones, glucagon, and catecholamines [5].

In hypopituitarism, GH deficiency and low cortisol due to adrenocorticotropic hormone (ACTH) deficiency result in increased insulin sensitivity and impaired gluconeogenesis, leading to recurrent hypoglycemia. Recurrent hypoglycemic episodes occur in patients with diabetes treated with insulin or SUs after hypophysectomy, pituitary apoplexy, or Sheehan syndrome, requiring careful adjustment of insulin and antidiabetic doses to prevent hypoglycemia [11]. Conversely, hyperglycemia is common in acromegaly, though hypoglycemia may indicate insulinoma or MEN 1 syndrome [12]. SSAs can disrupt the balance between GH, glucagon, and insulin, causing hyperglycemia or, less often, hypoglycemia [13]. Treatments like recombinant insulin-like growth factor-1(IGF-1), Mecasermin, for growth failure in children can also induce hypoglycemia [13].

In hypothyroidism, whether primary or secondary, hypoglycemia may occur due to associated hormone deficiencies or as part of autoimmune polyglandular failure. Blunted glycolysis and gluconeogenesis in muscle and adipose tissue result in delayed recovery from hypoglycemia. Impaired glucagon response and slowed gastric emptying are other contributing factors [14]. In myxedema coma blood glucose (BG) monitoring is critical for prevention and appropriate hypoglycemia treatment [14]. In Graves’ disease, AIH rarely occurs after methimazole. Additionally, the use of non-selective beta-blockers in patients with hyperthyroidism may result in masking early autonomic symptoms of hypoglycemia and delayed recovery from hypoglycemia [15].

In adrenal insufficiency or after adrenalectomy, reduced glucocorticoid secretion increases hypoglycemia risk [16]. Early post-adrenalectomy, pheochromocytoma patients may experience hypoglycemia due to a sudden drop in catecholamines, enhancing insulin secretion and suppressing glucagon [17]. Monitoring BG is critical in cases of adrenal crisis, after adrenalectomy due to pheochromocytoma or other causes, to prevent and manage hypoglycemia effectively.

### AASD position statement

- In patients with FH and hyperinsulinemia, insulinoma and factitious hypoglycemia should be considered and differentiated.
- In patients with diabetes and concurrent endocrinal insufficiency, care should be taken in prescribing antidiabetic drugs.
- In myxedema coma, during Addisonian crisis and after adrenalectomy, BG should be monitored and maintained.

### Non-diabetic Hypoglycemia in Liver Disease

**Fig. 2:**
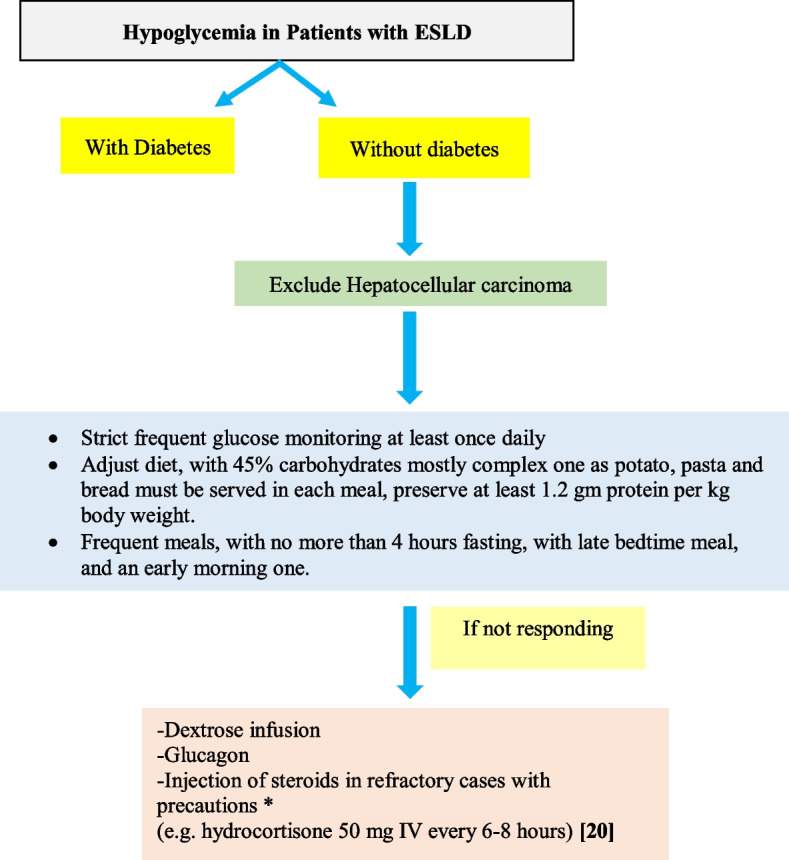
Algorithm demonstrating the approach to non-diabetic hypoglycemia in patients with ESLD

The liver is the largest metabolic organ that plays a pivotal role in glucose metabolism; it serves as a gatekeeper for maintaining and regulating BG in our bodies, mainly through glycogenolysis and gluconeogenesis. Therefore, any disruption of liver metabolism may decrease its efficacy in regulating BG levels [18], with a high incidence of hypoglycemia in patients with end-stage liver disease (ESLD) (Fig. 2), reaching up to 56% [18]. A survival analysis showed that hypoglycemia in cirrhotic patients with acute decompensated liver failure was significantly associated with lower estimated survival (36 days) compared to patients with normal blood sugar (54 days) [19].

The liver serves as a buffer for BG concentration. After 3–4 h of fasting, blood sugar decreases to 80-90 mg/dl, and the liver begins providing glucose through gluconeogenesis. During severe liver disease, it is difficult to maintain glycemic balance and to control BG fluctuations [18]. Essentially all stored glycogen in the liver is depleted after ~ 12 h of fasting. This depletion occurs more rapidly in liver diseases, such as after overnight fasting [20].

Patients with liver cirrhosis have a higher incidence of non-diabetic hypoglycemia because of damaged liver tissue in ESLD, acute-on-chronic liver failure, and malnutrition permanently associated with liver cirrhosis, where hypoglycemia serves as a clinical indicator [21]. Speculated contributing factors include compromised liver metabolic capacity, reduced food intake, glycogen depletion and gluconeogenesis disorders, and disruption of the inactivation of glucose-lowering hormones [21]. Additionally, severe septic infections in cirrhotic patients are associated with poor prognosis and high mortality when concomitant hypoglycemia is present at admission [22].

Islet cell and non-islet cell tumors can induce non-diabetic hypoglycemia, patients with non-islet cell tumors have an increased risk due to high metabolic requirements of the tumor cells, and the paraneoplastic syndrome [23]. Hepatocellular carcinoma (HCC) is the second most common cause of non-islet cell tumor hypoglycemia (NICTH), with an incidence reaching up to 27%. Hypoglycemia may even be the initial presentation of HCC due to paraneoplastic syndrome and/or the high metabolic demands and is associated with poor prognosis [23].

### AASD position statement

- Implement frequent and strict glucose monitoring (at least once daily), in patients with ESLD without diabetes.
- Adjust dietary composition to include 45% carbohydrates (mostly complex sources as potatoes, pasta and bread) in each meal, with at least 1.2 g of protein/kg of body weight.
- Consume frequent meals with no more than 4 h fasting intervals, including a late night and an early morning meal.
- Consider dextrose infusion, steroids or glucagon for hypoglycemia not responsive to nutritional modifications.

### Non-diabetic Hypoglycemia in Kidney Disease

Non-diabetic hypoglycemia in chronic kidney disease (CKD) occurs spontaneously in 1% −3% of patients [24]. End-stage renal disease (ESRD) requiring renal replacement therapy (RRT), along with factors such as advanced age, hospitalization, or intensive care unit (ICU) admission due to acute illness or infection, significantly increases condition complexity and prevalence [25].

**Fig. 3:**
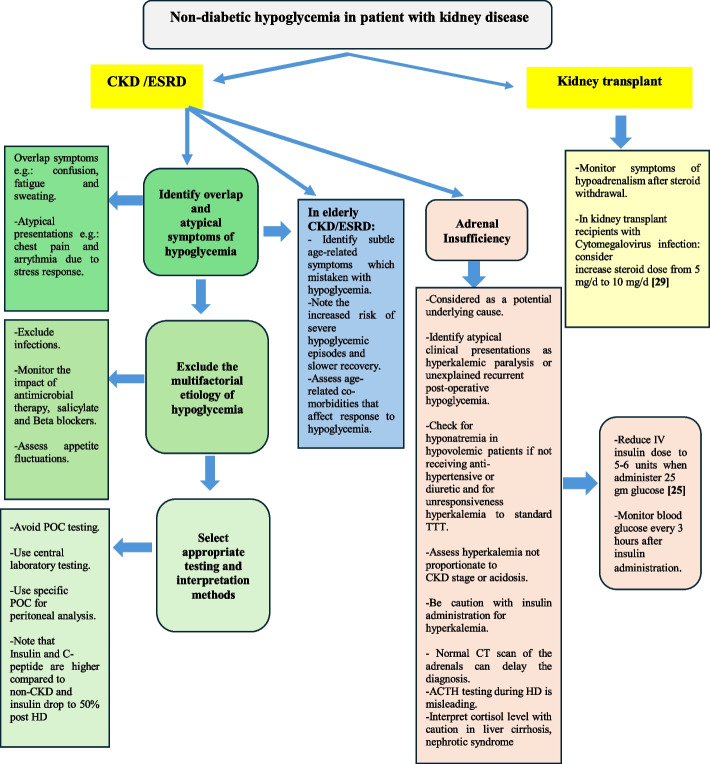
Algorithm demonstrating the approach to non-diabetic hypoglycemia in patients with kidney disease

Non-diabetic hypoglycemia is often underdiagnosed due to its low incidence and subtle presentation (Fig. 3), delaying its inclusion in differential diagnoses. CKD/ESRD patients exhibit atypical symptoms and responses to hypoglycemia. ESRD patients experience blunted counter-regulatory response, increasing severe episode risk [25], especially in the elderly, where subtle symptoms and slower recovery increase complication risks. Anxiety, irritability, and tremors are often less pronounced, while neuroglycopenic symptoms like confusion and memory loss predominate and may mimic dementia.

Diagnosing non-diabetic hypoglycemia in CKD/ESRD patients is challenging due to symptoms overlap with uremia or cardiovascular manifestations [25]. Atypical presentations include hyperkalemic paralysis or recurrent postoperative hypoglycemia or after partial nephrectomy. Additionally, normal adrenal CT scans may delay the diagnosis [26].

Non-diabetic hypoglycemia with hyponatremia in hypovolemic patients without antihypertensives or diuretics, or hyperkalaemia disproportionate to CKD stage, should prompt evaluation for adrenal insufficiency [27].

Laboratory interpretation for diagnosing non-diabetic hypoglycemia in CKD patients require caution due to variables like hypothermia, hypotension, anemia, pH changes, and hypoxia. To ensure accurate diagnosis, use central laboratory glucose measurements instead of point-of-care (POC) methods. ESRD patients using icodextrin for peritoneal dialysis require glucose-specific glucometers to avoid false readings from non-specific POC devices [25].

Kidney transplant receipts may develop hypoglycemia. Chronic low-dose prednisolone can suppress the hypothalamic–pituitary–adrenal (HPA) axis, causing adrenal insufficiency after withdrawal. There is no correlation between treatment duration and adrenal function tests [28]. Cytomegalovirus infection may also cause symptomatic hypoglycemia in kidney transplant recipients, improving with glucocorticoid dose escalation to 10 mg/day daily [29, 30].

Interpretation of insulin and C-peptide levels is complex in ESRD patients, as fasting levels are elevated compared to patients with normal kidney function, with insulin drop by 50% post- hemodialysis (HD), likely due to increased clearance [25]. ACTH testing during HD is unreliable due to altered metabolism and false low cortisol levels. Cortisol levels interpretation requires caution in clinical conditions like liver cirrhosis, hypoalbuminemia, or nephrotic syndrome [28]. Non-diabetic Hypoglycemia in ESRD patients is multifactorial and can be triggered by various events. Immunocompromised status increases infection-related hypoglycemic risk due to adrenal insufficiency or anti-microbial therapy [25, 30]. Dietary restrictions, changes in appetite, and polypharmacy (e.g., beta-adrenergic blockers and salicylates) can also contribute to BG fluctuations, resulting in the intermittent nature of hypoglycemia. Increased insulin sensitivity in ESRD patients necessitates care during medical treatment of hyperkalaemia. 13% of ESRD patients develop hypoglycemia with insulin administration during hypokalemia treatment, especially with adrenal insufficiency. Therefore, when administering the standard 25 g of dextrose parenterally (50 mL of 50% dextrose), the dose of intravenous insulin should be adjusted to 5–6 units of regular insulin, followed by close monitoring of BG levels hourly for 2–3 h post-administration [25].

### AASD position statement

- Maintain high suspicion for hypoglycemia in CKD/ESRD patients. Initiate treatment empirically during diagnostic workup.
- Understand appropriate methods and timing for hypoglycemia testing and its underlying causes.
- Multiple contributing factors often coexist in CKD/ESRD patients with non-diabetic hypoglycemia.
- Exclude Iatrogenic causes.
- Monitor glucose vigilantly in immunocompromised patients with additional risks (alcoholism, drug use, malnutrition, or long-term steroid use).

### Non-diabetic Hypoglycemia in Sepsis

**Fig. 4:**
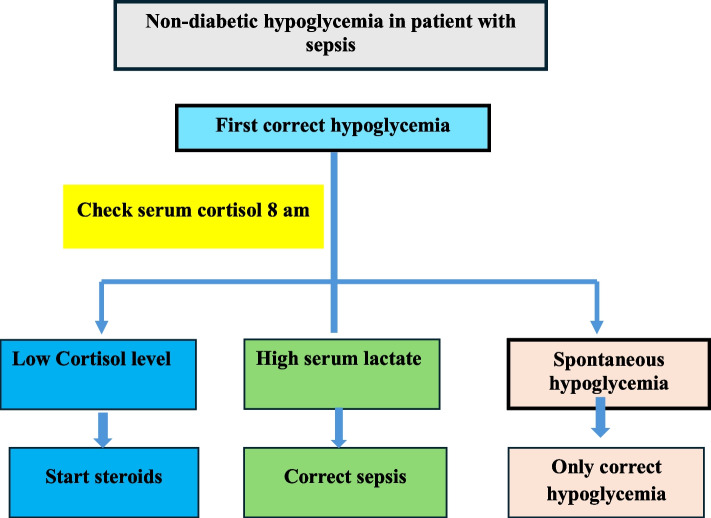
Algorithm demonstrating the approach to non-diabetic hypoglycemia in patients with sepsis

Changes in blood sugar levels have been observed in sepsis patients without diabetes (Fig. 4). These changes include stress hyperglycemia, spontaneous hypoglycemia, and glucose variability (GV), all associated with prolonged hospitalization, increased morbidity, and mortality [31]. The pathophysiologic mechanisms for sepsis-associated hypoglycemia (SAH) are multifactorial and include depletion of glycogen stores due to increased metabolic demands, impaired hepatic gluconeogenesis caused by inflammatory cytokines, excess lactate, and liver failure in the setting of multiple organ failure [32], sepsis- associated acute kidney injury resulting in impaired insulin catabolism and decreased tubular glucose absorption, and relative adrenal insufficiency is another possible underlying pathogenetic mechanism of SAH. The HPA axis is activated in sepsis to increase adrenal cortisol secretion but may be insufficient for demands. The inflammatory cytokines also impair the stimulatory effect of ACTH on adrenals, leading to “critical illness-related adrenocortical insufficiency (CIRCI)” which has been reported in 60% of patients with sepsis [33]. Early glucocorticoid administration corrects relative adrenal insufficiency with longer low-dose courses potentially improving long-term survival [33].

Notably, early sepsis hypoglycemia predicts increased mortality and prolonged hospitalization but remains excluded from the sequential organ failure assessment (SOFA) score [34].

### AASD position statement

- ICU physicians should recognize sepsis- hypoglycemia association, the impact of hypoglycemia on patient’s prognosis, and the pathogenic role of relative adrenal insufficiency.
- Administer glucocorticoids early in sepsis to address relative adrenal insufficiency.
- Implement strict BG monitoring to prevent and correct hypoglycemia promptly.
- Urgently investigate the utility of hypoglycemia as a sepsis severity predictor.

### Non-diabetic Hypoglycemia in Cancer

Non-islet cell tumor hypoglycemia (NICTH), also called *Doege-Potter syndrome,* is a rare but serious paraneoplastic syndrome [35]. The tumors are usually large, malignant, and either mesenchymal or epithelial in origin. NICTH occurs most commonly in patients with hepatocellular carcinoma (HCC) and less frequently, in those with colorectal carcinomas, fibromas, carcinoids, myelomas, and lymphomas. This type of hypoglycemia usually occurs due to tumor over production of incompletely processed pro-IGF-2 (rarely IGF-1). This results in insulin receptor stimulation, increased glucose utilization (especially by skeletal muscles), and suppression of hepatic glucose production via inhibited glycogenolysis/gluconeogenesis. Pro-IGF-2 also suppresses glucagon and GH release, aggravating hypoglycemia in NICTH [35].

Additional mechanisms include the production of insulin/insulin receptor autoantibodies, particularly in patients with multiple myeloma and Hodgkin lymphoma. Hypoglycemia may also result from extensive tumor burden replacing the hepatic tissue, with or without glucocorticoid deficiency caused by adrenal glands damage or hemorrhage or, rarely, insulin secretion by non-islet tumors [36]. Patients with NICTH usually present with the classic Whipple triad of hypoglycemia. Hypokalemia may occur in patients with IGF-2-secreting tumors due to the insulin-like action of pro-IGF-2 [37].

**Fig. 5:**
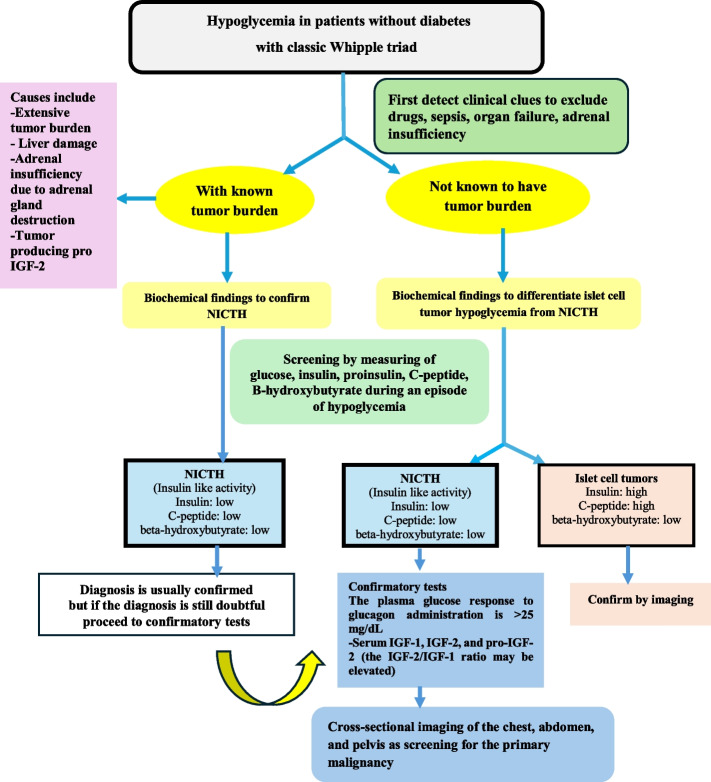
Algorithm demonstrating the approach to non-diabetic hypoglycemia in patients with cancer

Diagnosis of NICTH (Fig. 5) requires excluding other possible causes of hypoglycemia, such as pancreatic tumors, adrenal insufficiency, and drug-induced hypoglycemia. If clinical suspicion for NICTH is high, preliminary laboratory tests should include serum glucose, insulin, proinsulin, C-peptide, beta-hydroxybutyrate, and sulfonylurea/meglitinide screening during a hypoglycemia episode. Unlike patients with hyper insulinemic hypoglycemia, NICTH is characterized by low serum insulin, C-peptide level, and beta-hydroxy butyrate levels indicating insulin-like activity [37]. Plasma glucose response to glucagon administration more than 25 mg/dL can be used to confirm the insulin-like activity, unless there is massive hepatic tissue replacement by the tumor resulting in low hepatic glycogen stores [37].

If the diagnosis remains unconfirmed, measurement of serum IGF-1, IGF-2, pro-IGF-2 and elevated IGF-2/IGF-1 ratio may be helpful. However, normal IGF levels do not exclude the diagnosis if the hypoglycemia is non-IGF-mediated. For autoimmune NICTH, serum insulin and C-peptide levels are usually normal or elevated. Assessment of insulin auto-antibodies can confirm the diagnosis [37].

Treatment of NICTH focuses on correction of hypoglycemia and management of the underlying malignancy. The cornerstone of therapy for NICTH is cytoreduction of the underlying malignancy [36]. If the tumor is not resectable, palliative modalities such as tumor debulking, chemotherapy, radiotherapy, radiofrequency ablation, cryoablation or selective embolization of tumor feeding vessel may be used to control the tumor and improve hypoglycemia [37]. If the underlying malignancy is untreatable, medical therapy may be required to prevent recurrent hypoglycemia. The first choice is glucocorticoids (prednisone 30–60 mg daily) [38]. Other medical therapy includes glucagon, or recombinant human growth hormone (rhGH), however, its use has the potential to promote tumor growth [38].

### AASD position statement

- NICTH is a rare severe paraneoplastic syndrome diagnosed after excluding common causes of hypoglycemia.
- Cytoreduction of the underlying malignancy is the cornerstone of NICTH treatment, as hypoglycemia often recurs with tumor regrowth.
- For unresectable tumors, glucocorticoids are first-line medical therapy.

### Non-diabetic Reactive Hypoglycemia

Reactive hypoglycemia (RH) or post-prandial hypoglycemia (PPH) typically occurs 2–5 h postprandially. It is classified into 3 types based on timing and causes. *Alimentary RH or Late Dumping Syndrome (LDS)* occurs within 120 min after eating, commonly due to bariatric or non-bariatric stomach/oesophageal surgeries (e.g. fundoplication for reflux, partial gastrectomy, vagotomy). These surgeries result in rapid food delivery to the intestine causing exaggerated secretion of glucagon like peptide-1 (GLP-1) and gastrointestinal peptide (GIP) leading to excessive insulin secretion and glucagon secretion suppression [39].

*Late RH,* often in individuals with prediabetes, occurs 240–300 min post-meal due to delayed insulin secretion and insulin resistance. This increases the risk of type 2 diabetes, even in normal weight individuals. Oral glucose tolerance test (OGTT) shows BG < 55 mg/dL at 4–5 h, and those individuals have family history of T2DM or obesity [39]. *Idiopathic RH* typically occurs about 3 h after eating. Although its exact mechanism remains unclear, it is believed to be linked to increased insulin sensitivity [39, 40].

Hormonal causes of RH include adrenal failure, hypothyroidism, and insulinoma, which typically causing fasting hypoglycemia and rarely RH. Nesidoblastosis mimics insulinoma with similar laboratory profiles but with negative imaging [40]. Rare possible causes include fructose intolerance and galactosemia [39].

RH is often triggered by excessive intake of high-glycemic index carbohydrates or simple sugars. These cause a spike in BG followed by excess insulin secretion, leading to a rapid BG drop and symptoms like fatigue, dizziness, hunger, and sugar cravings [41]. Contributing factors include inadequate glucagon response, alcohol and poor dietary habits. While BG correction often occurs without medical intervention, recurrent symptoms may indicate presence of an underlying condition requiring treatment [39]. Dietary management plays a fundamental role in RH, as the type and timing of food consumption affect BG levels. Avoiding simple carbohydrates, refined sugars, and alcohol while increasing fiber reduces hypoglycemic episodes. It is advisable to treat RH cautiously like other acute hypoglycemia; avoid overtreatment to prevent insulin excess and recurrent hypoglycemia cycles [40].

**Fig. 6:**
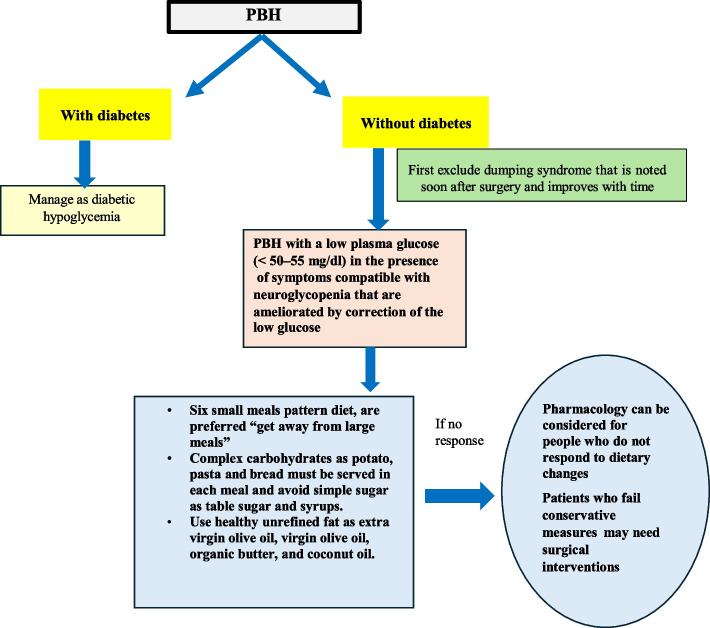
Algorithm demonstrating the approach to non-diabetic post-bariatric surgery hypoglycemia

Post-bariatric surgery hypoglycemia (PBH) (Fig. 6) is a serious complication of bariatric surgery with varying frequencies based on the procedure type, diagnostic methods, and BG thresholds. It is most common after Roux-en-Y Gastric Bypass (RYGB), less common after sleeve gastrectomy (SG), and rare after adjustable gastric banding (AGB). Asymptomatic cases have also been reported, complicating the accurate estimation of PBH frequency [42]. PBH significantly impacts the quality of life. Risk factors include female sex, younger age, pre-surgery diabetes, low pre-surgical A1c, and excessive weight loss. Symptoms range from autonomic overactivity to neuroglycopenic issues such as dizziness, confusion, or coma. Hypoglycemia unawareness, due to impaired counter-regulatory hormonal responses, can lead to only neuroglycopenic symptoms, which may be misdiagnosed as neurologic or psychiatric disorders [42].

**Fig. 7:**
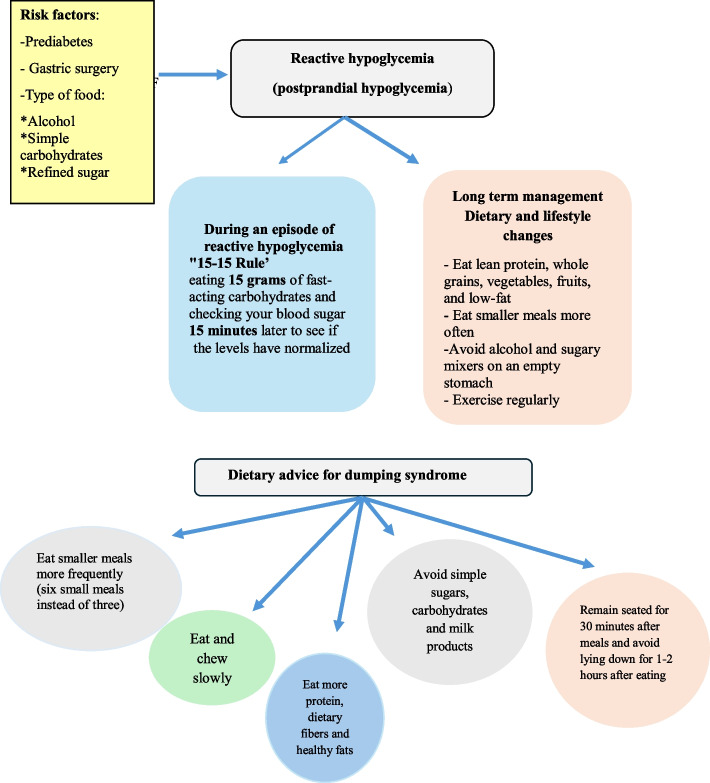
Algorithm demonstrating the management of reactive hypoglycemia and dumping syndrome

Diagnosing PBH is challenging due to several factors. First, there is no definitive BG cutoff for PBH. While BG < 60 mg/dL during OGTT or mixed meal tolerance test (MMTT) is commonly used, some experts accept lower thresholds [43]. Second, PBH can be confused with early dumping syndrome (EDS) (Fig. 7). PBH typically occurs 1–3 years after BS and persists for long-term, whereas EDS manifests soon after BS and improves over time. EDS symptoms arise 10–30 min post-meal due to rapid food transit which increases intestinal osmolarity and shifts fluid from intravascular compartment to the gut, causing abdominal discomfort, nausea, and vasomotor symptoms such as sweating, palpitation, flushing, dizziness and rarely syncope without hypoglycemia [44]. In contrast, PBH symptoms, such as reactive hypoglycemia, occur 1–3 h postprandial and are triggered by excessive rapidly absorbed carbohydrates, alcohol, or caffeine. In some patients, symptoms may occur nocturnally but not during fasting. Third, no gold standard diagnostic test exists for PBH, though the 180-min OGTT, MMTT, and 3-day CGM are primarily relied upon for diagnosis. PBH is also associated with glycemic variability, with an early postprandial BG spike followed by hypoglycemia, which further complicates the diagnosis [43].

PBH is a complex condition influenced by weight loss, increased insulin sensitivity, heightened β-cell response, surgical alterations, excessive secretion of GIP and GLP-1, impaired hormone responses, altered gut microbiota, bile acids, mucosal adaptations, and rapid gastric emptying. Nesidioblastosis is controversial [44–46].

PBH patients with mild to moderate symptoms often benefit from nutritional therapy, which aims to stabilize BG levels and slow intestinal absorption. This involves consuming six small solid meals daily, each containing limited complex carbohydrates (up to 30 g/meal) with a low glycemic index, adequate protein (0.91 g/kg of body weight/day), and soluble fiber. Heart-healthy fats such as olive oil, nuts, and avocado (15 g/meal, 5 g/snack) are included to provide calories while restricting carbohydrates. Liquids are avoided during meals and for 30 min after meals to prevent rapid glucose spikes. Simple sugars, refined carbohydrates, caffeine, and alcohol are excluded due to their potential to cause hypoglycemia. Increased fiber intake helps slow food transit and absorption [47, 48]. If nutritional therapy fails, pharmacological options like acarbose (which decreases glucose intestinal absorption), diazoxide, verapamil (decrease insulin secretion), or SSAs (which reduce insulin secretion and delay gastric emptying) may be used. Experimental treatments include glucagon pumps, GLP-1 receptor antagonists, and monoclonal antibodies. Surgery, such as partial pancreatectomy or laparoscopic reversal of RYGB and gastric banding in SG, is considered for refractory cases [48].

Management of RH due to other causes primarily involves dietary and behavioral changes [49]. A diet rich in whole grains, lean protein, vegetables, fruits, and low-fat foods, along with smaller more frequent meals, can reduce RH episodes. Patients should avoid alcohol, sugary drinks on an empty stomach, and should be advised to lie down for 1–2 h after eating. Chewing slowly and remaining seated for 30 min post-meals is also advised [49]. Dietary treatment for RH mirrors PBH. In severe cases, medications like octreotide, anti-diarrheal, pectin, or guar gum may be used. For late RH with prediabetes, metformin, acarbose, thiazolidinediones, sodium glucose transporter 2 (SGLT2) inhibitors, dipeptidyl peptidase (DPP-4) inhibitors, or GLP-1 receptor agonists may be prescribed on an individualized basis [48].

### AASD position statement

- Counsel BS candidates about PBH symptoms, its impact on quality of life, and available treatment options.
- RH is less common after non-bariatric upper gastrointestinal surgeries compared to BS, though they share similar mechanisms.
- Dietary intervention is the first-line treatment for RH. Medical therapy is for non-responders, and surgery is reserved for refractory cases.
- Screen for prediabetes in individuals with late RH.
- Conduct long-term studies to assess PBH sequelae and treatment safety and efficacy.

### Non-diabetic Hypoglycemia during Pregnancy

Non-diabetic hypoglycemia in pregnancy presents unique challenges in both diagnosis and management. Recognizing and treating hypoglycemia in pregnant patients is essential because of the potential risks to both the mother and the fetus.

Increased insulin sensitivity in early pregnancy results in lower-than-normal fasting blood sugar. Furthermore, capillary BG levels are higher than venous BG levels. Understanding these facts are critical to avoid misinterpreting BG results and prevent unnecessary interventions or missed opportunities for treatment [50].

Pregnant women may experience atypical hypoglycemic symptoms. Typical symptoms of hypoglycemia, such as sweating, shakiness, and confusion, may be mistaken for common pregnancy-related conditions such as morning sickness or pregnancy-related anxiety. Severe symptoms such as seizures or unconsciousness may be misattributed to other conditions, such as eclampsia or epilepsy. Hypoglycemia unawareness in some pregnant women could lead to missed or delayed diagnosis and treatment until severe complications arise, e.g., fetal distress or maternal coma [3]. In pregnant women who experience hypoglycemia, BG monitoring, preferably CGM, is essential for early detection and appropriate management. However, the cost of CGM can be a limiting factor.

Standard treatment protocols for hypoglycemia, such as the intake of simple carbohydrates followed by complex carbohydrates/proteins, may not always be suitable in pregnancy, as they might not account for the altered gastric emptying times and increased metabolic demands. Additionally, pregnant women without diabetes may require different management strategies than those with diabetes [51]. Over-reliance on standard protocols without considering individual related factors can lead to ineffective treatment or exacerbation of symptoms. Moreover, medications used for treating hypoglycemia, like glucagon, may exhibit altered pharmacokinetics during pregnancy, potentially affecting their efficacy [52].

Nutritional counseling is a critical component but is often underutilized or non-individualized. Pregnant women need a balanced intake of macronutrients. Without adequate guidance, women may either over-restrict carbohydrates to avoid blood sugar spikes or consume excessive simple sugars, leading to rebound hypoglycemia [53]. Hypoglycemia can also cause significant psychological stress. Women may experience anxiety about potential episodes, which can result in abnormal eating behavior and disrupt their quality of life [54].

### AASD position statement

Strategies to mitigate pitfalls in diagnosis and management require a multi-faceted approach including:

- Doctors and healthcare providers must understand the physiological changes in BG levels during pregnancy and how to accurately interpret these results
- Reliance on capillary blood glucose measurements alone is not sufficient. Venous BG measurement is the most accurate method for making treatment decisions.
- Nutritional counseling tailored to the needs of pregnant women, along with frequent glucose monitoring, are essential for creating an effective management plan. Dieticians specializing in pregnancy-related metabolic conditions can provide tailored dietary recommendations.
- Mental health support should be an integral part of hypoglycemia management. Counseling services and support groups should be offered to help women maintain their emotional well-being during pregnancy.

### Non-diabetic Hypoglycemia in Pediatrics

**Fig. 8:**
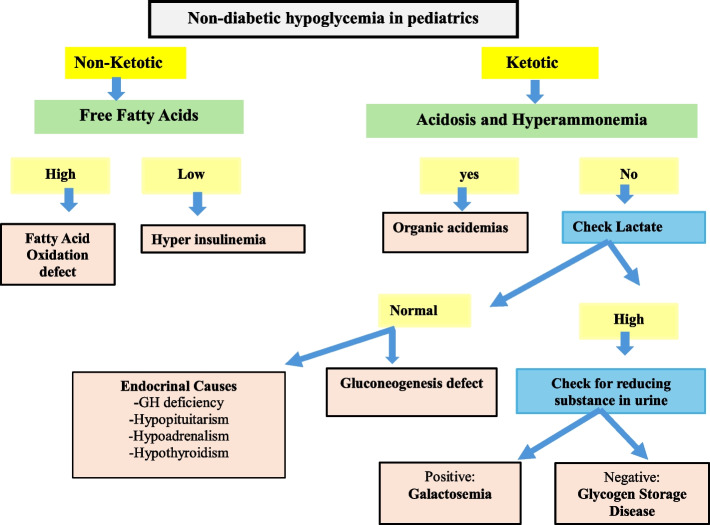
Algorithm demonstrating the approach to non-diabetic hypoglycemia in pediatrics

Hypoglycemia in children without diabetes presents unique challenges and pitfalls for clinicians, especially since the underlying causes, clinical presentation, and management vary significantly compared to children with diabetes (Fig. 8) [55].

Challenges in diagnosis include a broad differential diagnosis (e.g., hyperinsulinism, adrenal insufficiency, glycogen storage diseases) and nonspecific symptoms like irritability, fatigue, and headache, which may be mistaken for common pediatric conditions [55–57]. False positive and negative results are common when glucometers are used; therefore, laboratory assessment is critical. Differentiating between benign transient hypoglycemia (common in neonates) and persistent hypoglycemia due to underlying disorders can be difficult [55–57]. Certain types of hypoglycemia, such as ketotic hypoglycemia, often resolve as the child grows; however, diagnosis is usually made by exclusion [56]. Accurate diagnosis requires drawing a critical sample during a hypoglycemic event. In some cases, fasting the child for long hours to provoke hypoglycemia is necessary [55–57].

Diagnosing the underlying endocrine or metabolic cause often requires specialized tests (e.g., insulin levels, cortisol levels, metabolic profiles) which may not be readily available.

Some types of hypoglycemia linked to inborn errors of metabolism can only be confirmed with genetic testing, which may be difficult to access, particularly in low-resource settings [58].

Recognizing and treating acute hypoglycemic episodes promptly is critical to prevent seizures and brain injury, and, in severe cases, death [59]. Persistent non-diabetic hypoglycemia in children can be distressing for families, requiring constant vigilance and dietary adjustments, which may impact family dynamics and the child’s quality of life. Over-treating hypoglycemia with unnecessary carbohydrate supplements or dietary changes can lead to poor eating habits and unnecessary weight gain. On the other hand, under-treatment due to misinterpretation of symptoms or mild hypoglycemia can result in adverse neurodevelopmental outcomes [60]. Providing families with clear, concise guidelines is vital [60].

### AASD positional statement

- Evaluation of non-diabetic hypoglycemia in pediatrics should involve a team of specialists, including endocrinologists, pediatricians, and dietitians.
- Effective use of genetic and metabolic testing can help identify underlying disorders and guide precise treatment.
- The adoption of standardized diagnostic protocols and established guidelines is essential for accurate diagnosis and management.
- Targeted and balanced management plans should be followed, and new therapies, such as diazoxide for hyperinsulinism or specialized diets for metabolic disorders, should be incorporated into treatment strategies.
- A combination of standardized protocols, family education, specialist support, and close follow-up, can mitigate many pitfalls and lead to better outcomes.

### Neuropsychiatric manifestations of non-diabetic hypoglycemia

**Fig. 9:**
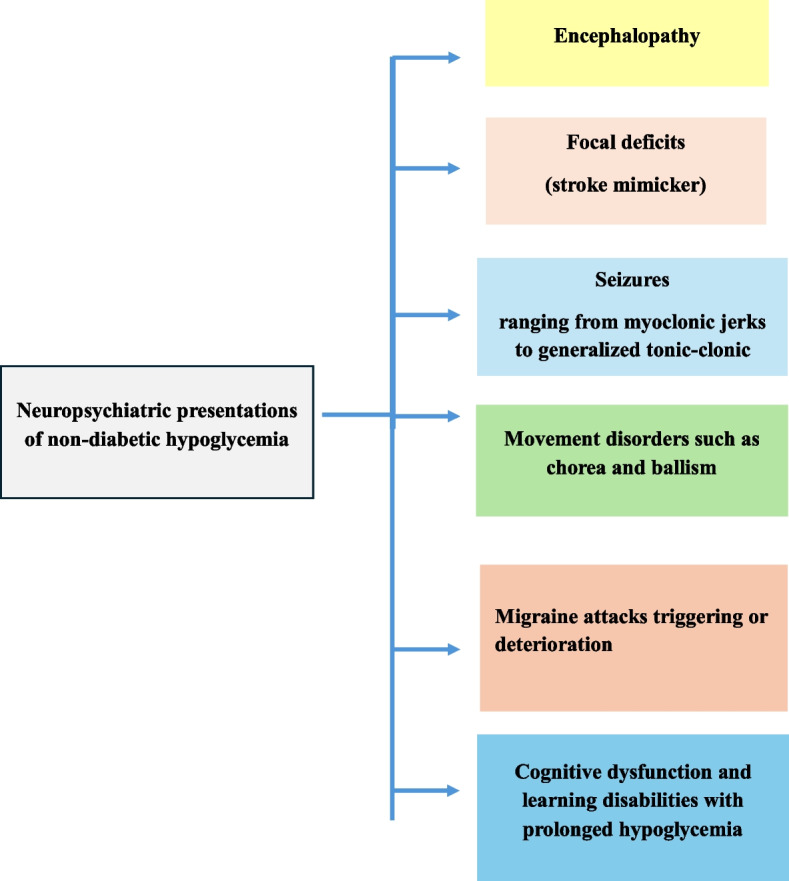
Neuropsychiatric manifestations of non-diabetic hypoglycemia

Neuroglycopenia, a shortage of glucose (glucopenia) in the brain, alters brain function and behavior (Fig. 9). Mild hypoglycemia causes pale skin, recurrent sweating, hunger, and associated sympathetic symptoms including palpitations, tremor, and anxiety. With prolonged duration or severity of symptoms, focal deficits including hemiplegia, aphasia, hemianopia, and cortical blindness can occur. Hypoxic injury to the basal ganglia can result in akinetic rigid states and symmetric parkinsonism. Seizures can range from generalized seizures to myoclonic jerks. Severe cases lead to decerebrate posturing, lethargy, vegetative states, and coma [61]. Prolonged or recurrent neuroglycopenia can result in permanent brain damage and death [4].

Hypoglycemia should be part of the differential diagnosis of patients presenting with clinical or imaging features of stroke or transient ischemic attack (TIA). Less severe episodes can cause hemiparesis and may mimic ischemic stroke. Alternatively, hypoglycemia can cause lesions in a wide range of brain regions but only those in the corticospinal tracts cause focal neurological symptoms (hemiparesis or hemisensory loss) sufficient to trigger scanning because of clinical similarity to stroke [62]. Acute tetraplegia has been rarely reported [63].

Differentiating a hypoglycemia-induced headache from a migraine is a clinical challenge, especially in a patient with a history of migraine, as low BG levels can trigger or worsen migraine attacks [61, 64].Acute symptomatic seizures can occur during hypoglycemia, as excitatory amino acids increase out of proportion to the slight rise in inhibitory GABA, creating a potential epileptic focus. Generalized tonic–clonic seizures have been observed [61].

Movement disorders such as chorea and ballism have been reported. Structures commonly involved include the cortex, corona radiata, hippocampus and basal ganglia. Sudden jerky head or leg movements may occur, possibly due to the involvement of the posterior limb of the internal capsule [65]. Hypoglycemia can also affect cognition, including memory, language, attention, and visuospatial orientation [61].

### AASD position statement

- Hypoglycemia should be considered as a potential cause of any alteration in consciousness.
- Patients with neuropsychiatric manifestations must be rapidly screened for hypoglycemia, with a bedside determination of the BG level, and treated appropriately, regardless of presumptive diagnoses.
- Significant irreversible central nervous system damage may occur if the BG concentration is not rapidly corrected.
- Prompt normalization of mental status via dextrose infusion may prevent the need for more invasive interventions like neuromuscular blockade-assisted intubation.

### Medication-related Nondiabetic Hypoglycemia

Studies suggest that non-diabetic hypoglycemia may affect around 1% of the general population [49]. However, this number may be higher, as many cases go unreported or undiagnosed. Medication-related hypoglycemia is a leading etiology of non-diabetic hypoglycemia [66]. Therefore, a meticulous medication review of over-the-counter (OTC) and prescription medications is essential. These medications contribute to hypoglycemia through various mechanisms, such as stimulating insulin release, reducing its clearance, altering sensitivity to insulin or interfering with glucose metabolism [67].

A list of medications associated with non-diabetes hypoglycemia is provided in Table (1).

**Table 1:** Medications associated with non-diabetic hypoglycemia

Nonsteroidal anti-inflammatory drugs (NSAIDs) (e.g., ibuprofen, naproxen) Indomethacin: analgesic Tricyclic antidepressants (TCAs): (e.g., doxepin, imipramine, nortriptyline) Antidepressant of selective serotonin re-uptake inhibitors (SSRIs) family: (e.g., fluoxetine, sertraline) Mono-Amin Oxidase Inhibitors (MAOIs): (e.g., selegiline, rasagiline) Antipsychotics, lithium and valproate Certain antibiotics (e.g., gatifloxacin, levofloxacin, sulfamethoxazole) Antimalarial drugs (e.g., quinine, chloroquine) Certain antiviral drugs (e.g., pentamidine) Angiotensin converting enzyme inhibitors (ACEI) (e.g., lisinopril, enalapril) Quinine, cibenzoline and disopyramide (used for heart arrhythmias) Mifepristone: a progesterone blocker Factitious intake of insulin, sulfonylureas, and other diabetes medicines Beta-blockers: these medications can mask the symptoms of hypoglycemia, making it more difficult to diagnose and treat	

### AASD position statement

- Meticulous medication history review, including OTC drugs, is a crucial initial step in the assessment of hypoglycemia in patients without diabetes. This step can save time and costs by avoiding unneeded investigations.

### Limitations

This consensus statement has several noteworthy limitations. First, the scope of our review did not encompass the complex interactions between diabetes and hypoglycemia, which have been extensively studied elsewhere. Second, while we conducted a comprehensive literature review, we could not cover all existing research not captured by our search engines. Additionally, our attempt to provide recommendations for Arab countries may oversimplify the significant healthcare variations across the region. Finally, as a consensus statement rather than a meta-analysis, our methodology did not include quantitative data synthesis, which limits our ability to draw statistically robust conclusions. These limitations highlight the need for future research to address these gaps.

## Conclusion

Non-diabetic hypoglycemia is a rare phenomenon, as counter-regulatory mechanisms in healthy individuals typically prevent and correct it. It is not a diagnosis but a manifestation of an underlying disorder. The evaluation and management of non-diabetic hypoglycemia are challenging and require a comprehensive and balanced approach, including a detailed history, meticulous medication review, and a thorough physical exam to guide the diagnostic process in patients with documented hypoglycemia. Therefore, common pitfalls in diagnosis and management should be well understood to minimize unnecessary evaluations and costs and, conversely, to avoid misinterpreting or underdiagnosing patients with treatable or serious underlying disorders.

## Data Availability

No datasets were generated or analysed during the current study.

## Abbreviations

AASD: The Arabic Association for the Study of Diabetes and Metabolism
ACEI: Angiotensin converting enzyme inhibitors
ACTH: Adrenocorticotrophic hormone
AGB: Adjustable gastric banding
AIH: Autoimmune hypoglycemia
BS: Bariatric surgery
BG: Blood glucose
CGM: Continuous glucose monitoring
CIRCI: Critical illness-associated adrenocortical insufficiency
CKD: Chronic kidney disease
CT: Computed tomography
DDP-4: Dipeptidyl peptidase-4
EDS: Early dumping syndrome
ESLD: End-stage liver disease
ESRD: End-stage renal disease
FH: Fasting hypoglycemia
GABA: Gamma-aminobutyric acid
GH: Growth hormone
GIP: Gastrointestinal peptide
GLP: Glucagon-like peptide
GV: Glucose variability
HCC: Hepatocellular carcinoma
HD: Hemodialysis
HOMA-IR: Homeostatic Model Assessment for Insulin Resistance
IAS: Insulin autoimmune syndrome
ICU: Intensive care unit
IGF: Insulin-like growth factor
LDS: Late dumping syndrome
MAOIs: Mono-Amin Oxidase Inhibitors
MEN: Multiple endocrine neoplasia
MMTT: Mixed meal tolerance test
MRI: Magnetic resonance imaging
NICTH: Non-islet cell tumor hypoglycemia
NSAIDs: Nonsteroidal anti-inflammatory drugs
OGTT: Oral glucose tolerance test
OTC: Over the counter
PBH: Post-bariatric-surgery hypoglycemia
PPH: Postprandial hypoglycemia
POC: Point-of-care
RH: Reactive hypoglycemia
rhGH: Recombinant human growth hormone
RRT: Renal replacement therapy
RYGB: Roux-en-Y gastric bypass
SAH: Sepsis associated hypoglycemia
SG: Sleeve gastrectomy
SGLT 2: Sodium glucose transporter 2
SSA: Somatostatin analogues
SSRIs: Selective serotonin re-uptake inhibitors
SOFA: Sequential organ failure assessment
SU: Sulfonylurea
T2DM: Type 2 diabetes mellitus
TCAs: Tricyclic antidepressants
TIA: Transient ischemic attack
